# Little Things

**Published:** 1871-04

**Authors:** 


### LITTLE THINGS.

In addition to the ordinary use of rubber tubes for moving irregular teeth, I occasionally find a narrow ring very useful in guarding from moisture cavities on the lingual and buccal surfaces which approach closely or pass the gum margin. The shape of a tooth, when an elastic ring is passed over the crown, favors movement toward the gum, and thus exudations which would be very troublesome are restrained by the the compress.

Not long since a patient called, suffering intense pain in a lower cuspid or bicuspid, which had the pulp slightly exposed by abrasion of plate, or shallow decay below the crown, on the posterior surface. There was no adjoining tooth on that side, and there was no cavity to hold cotton. How could I retain there any agent to allay pay? Especially how could so dangerous an agent as arsenic be used there? I thought of my rubber tube; cut off a ring wide enough to cover the decayed portion of tooth, passed it over the crown, and in a moment had a complete pocket to retain applications of either creosote or arsenic safely. PRACTICE.

